# Cytokine Profile in Striated Muscle Laminopathies: New Promising Biomarkers for Disease Prediction

**DOI:** 10.3390/cells9061532

**Published:** 2020-06-23

**Authors:** Cristina Cappelletti, Irene Tramacere, Paola Cavalcante, Elisa Schena, Luisa Politano, Nicola Carboni, Alessandra Gambineri, Adele D’Amico, Lucia Ruggiero, Giulia Ricci, Gabriele Siciliano, Giuseppe Boriani, Tiziana Enrica Mongini, Liliana Vercelli, Elena Biagini, Matteo Ziacchi, Maria Rosaria D’Apice, Giovanna Lattanzi, Renato Mantegazza, Lorenzo Maggi, Pia Bernasconi

**Affiliations:** 1 Neurology IV-Neuroimmunology and Neuromuscular Diseases Unit, Fondazione IRCCS Istituto Neurologico Carlo Besta, 20133 Milan, Italy; paola.cavalcante@istituto-besta.it (P.C.); renato.mantegazza@istituto-besta.it (R.M.); lorenzo.maggi@istituto-besta.it (L.M.); pia.bernasconi@istituto-besta.it (P.B.); 2 Department of Research and Clinical Development, Fondazione IRCCS Istituto Neurologico “Carlo Besta”, 20133 Milan, Italy; irene.tramacere@istituto-besta.it; 3 CNR Institute of Molecular Genetics, Unit of Bologna, 40136 Bologna, Italy; elisaschena83@gmail.com (E.S.); giovanna.lattanzi@cnr.it (G.L.); 4 Endocrinology Unit, Department of Medical & Surgical Sciences, Alma Mater Studiorum University of Bologna, S Orsola-Malpighi Hospital, 40138 Bologna, Italy; 5 Cardiomyology and Medical Genetics, Department of Experimental Medicine, University of Campania, Luigi Vanvitelli, 80138 Naples, Italy; luisa.politano@unicampania.it; 6 Neurology Department, Hospital San Francesco of Nuoro, 08100 Nuoro, Italy; nikola.carboni@tiscali.it; 7 Endocrinology Unit, Department of Clinical and Medical Science, S. Orsola-Malpighi Hospital, University of Bologna, 40138 Bologna, Italy; alessandra.gambiner3@unibo.it; 8 Unit of Neuromuscular and Neurodegenerative Disorders, Bambino Gesù Children’s Hospital, 00165 Rome, Italy; adeledamico@hotmail.com; 9 Department of Neurosciences and Reproductive and Odontostomatologic Sciences, University Federico II, 80137 Naples, Italy; lucia.ruggiero@unina.it; 10 Department of Clinical and Experimental Medicine, Neurological Clinic, 56126 Pisa, Italy; g_ricci@alice.it (G.R.); g.siciliano@med.unipi.it (G.S.); 11 Cardiology Division, Department of Biomedical, Metabolic and Neural Sciences, University of Modena and Reggio Emilia, Policlinico di Modena, 41121 Modena, Italy; giuseppe.boriani@unimore.it; 12 Department of Neurosciences “Rita Levi Montalcini”, University of Turin, 10124 Turin, Italy; tizianaenrica.mongini@unito.it (T.E.M.); lilianavercelli@hotmail.com (L.V.); 13 Azienda Ospedaliero Universitaria - Policlinico di St. Orsola, Cardiology Unit, Cardio-Thoracic-Vascular Department, 40138 Bologna, Italy; elena.biagini@aosp.bo.it (E.B.); matteo.ziacchi@aosp.bo.it (M.Z.); 14 Medical Genetics Unit, Policlinico Tor Vergata University Hospital, 00133 Rome, Italy; d.apice@med.uniroma2.it; 15 IRCCS Istituto Ortopedico Rizzoli, 40136 Bologna, Italy

**Keywords:** cytokines, laminopathies, macrophages, muscle damage, skeletal muscle

## Abstract

Laminopathies are a wide and heterogeneous group of rare human diseases caused by mutations of the LMNA gene or related nuclear envelope genes. The variety of clinical phenotypes and the wide spectrum of histopathological changes among patients carrying an identical mutation in the LMNA gene make the prognostic process rather difficult, and classical genetic screens appear to have limited predictive value for disease development. The aim of this study was to evaluate whether a comprehensive profile of circulating cytokines may be a useful tool to differentiate and stratify disease subgroups, support clinical follow-ups and contribute to new therapeutic approaches. Serum levels of 51 pro- and anti-inflammatory molecules, including cytokines, chemokines and growth factors, were quantified by a Luminex multiple immune-assay in 53 patients with muscular laminopathy (Musc-LMNA), 10 with non-muscular laminopathy, 22 with other muscular disorders and in 35 healthy controls. Interleukin-17 (IL-17), granulocyte colony-stimulating factor (G-CSF) and transforming growth factor beta (TGF-β2) levels significantly discriminated Musc-LMNA from controls; interleukin-1β (IL-1β), interleukin-4 (IL-4) and interleukin-8 (IL-8) were differentially expressed in Musc-LMNA patients compared to those with non-muscular laminopathies, whereas IL-17 was significantly higher in Musc-LMNA patients with muscular and cardiac involvement. These findings support the hypothesis of a key role of the immune system in Musc-LMNA and emphasize the potential use of cytokines as biomarkers for these disorders.

## 1. Introduction

The lamin A and C proteins, produced through the alternative splicing of the LMNA gene on chromosome 1, are type V intermediate filaments located in the nuclear lamina, beneath the inner nuclear membrane [1,2,3]. Mutations of the LMNA gene cause a wide and heterogeneous group of diseases defined as laminopathies, which may affect skeletal and cardiac muscle, bone, adipose tissue and peripheral nerves and may be associated with accelerated aging [4].

The variety of clinical phenotypes and the wide spectrum of histopathological changes among patients carrying an identical mutation of the LMNA gene make the diagnostic process difficult for laminopathies [5], and classical genetic screens appear to have limited predictive value for disease development. Clinical outcome measures have been poorly investigated in muscular laminopathies (Musc-LMNA) and natural history has not been elucidated yet. Hence, the availability of objectively measurable, easily obtainable and reproducible circulating biomarkers would be an extremely useful tool to help differentiate and stratify disease subgroups, which would aid in the evaluation of disease progression in clinical practice and in pharmacological clinical trials; furthermore, circulating biomarkers may represent possible therapeutic targets in Musc-LMNA.

A complex proteomic analysis of plasma samples derived from LMNA-mutated patients identified several proteins, mainly cytokines, related to inflammatory response and homeostasis maintenance [6]. Many of them belonged to networks related to stress response or negative regulation of apoptosis and were characterized by a signal peptide for the classical secretory pathway. A significant amount of the proteins identified were derived from an ER/Golgi-independent protein secretion pathway [6]. Furthermore, in Musc-LMNA, recent findings demonstrated the presence of few discernible immune cells infiltrating muscle tissue [7]. Indeed, besides a few CD11c^+^ myeloid dendritic cells, CD4^+^ T lymphocytes and rare CD138^+^ plasma cells, the majority of mononuclear immune cells have been shown to be composed by CD68^+^ macrophages. CD68^+^ macrophages are considered a major source of many cytokines involved in immune responses, hematopoiesis, inflammation and many other homeostatic processes [8]. Indeed, upon stimulation, macrophages synthesize and release a large variety of pro- and anti-inflammatory cytokines, including interleukin-1β (IL-1β), interleukin-6 (IL-6), interleukin-8 (IL-8), interleukin-12 (IL-12), transforming growth factor beta (TGF-β), granulocyte-macrophage colony-stimulating factor (GM-CSF) and tumor necrosis factor-alpha (TNF-α) [9].

Although cytokines are involved in various disease states and are dependent upon different factors, several studies demonstrated the possibility of using these molecules as a diagnostic and prognostic tool for various pathological conditions with greater ease and accuracy and at a lower cost [10,11,12,13]. 

The purpose of the present study was to use a comprehensive immune-profiling approach to determine whether an abnormal profile of circulating cytokines could be identified in Musc-LMNA patients, and whether this profile may distinguish the form of laminopathy from other types of muscular disorders or differentiate from distinct non-muscular phenotypes caused by LMNA mutations.

## 2. Materials and Methods

### 2.1. Patients and Healthy Controls

In this study, two groups of patients have been included. The first group consisted of 40 Musc-LMNA patients (15 females and 25 males, mean age at blood collection ± standard deviation (SD): 43.76 ± 16.80 years), including 11 with limb-girdle muscular dystrophy type 1B (LGMD1B), 17 with Emery–Dreifuss muscular dystrophy 2 (EDMD2) and 12 LMNA patients with cardiac conduction disorders (LMNA-CCD) or with dilated cardiomyopathy (LMNA-DCM). Ten patients had a mutated LMNA gene without skeletal muscle involvement (hereinafter called LMNA-mutated) including 3 who had mandibuloacral dysplasia with type A lipodystrophy (MADA), 1 with Dunnigan-type familial partial lipodystrophy (FPLD2), 4 asymptomatic patients and 2 neuropathic patients (5 females and 5 males, mean age at blood collection ± SD: 36.17 ± 15.41 years). Twenty-two patients were affected by other muscular diseases (3 females and 19 males, mean age at blood collection ± SD: 32.5 ± 14.24 years), including 3 with Duchenne muscular dystrophy, 3 with Becker muscular dystrophy, 3 with myotonic dystrophy type 1, 11 who had Emery–Dreifuss muscular dystrophy 1 (EDMD1), 1 with idiopathic dilated cardiomyopathy and 1 with familiar dilated cardiomyopathy. These were compared with 22 age- and sex-matched healthy controls (19 females and 3 males, mean age at blood collection ± SD: 30.73 ± 7.99 years (Table 1)). The second group, selected to quantify the concentration of some of the most relevant TNF-related cytokines, included 13 additional Musc-LMNA patients (6 females and 7 males, mean age at blood collection ± SD: 49.18 ± 12.86 years) and 15 new healthy controls (9 females and 6 males, mean age at blood collection ± SD: 41.25 ± 7.94 years). 

Laminopathic patients may show cardiac and/or muscle involvement at different stages of the disease. In this study, the Musc-LMNA patients were grouped based on the presence of: (1) only myopathy; (2) only cardiomyopathy (dilated cardiomyopathy and/or arrhythmia); (3) myopathy plus cardiomyopathy.

All patients or their guardians and controls signed an informed consent form for use of their biological samples and clinical data for research purposes in an anonymized form.

### 2.2. Serum Sample Collection

The peripheral blood of the Musc-LMNA and LMNA-mutated patients, as well as the healthy controls, was drawn between 8:00 a.m. and 4:00 p.m. on the day of inclusion into the study. For patients with other muscular diseases, sera samples were provided by the Naples Human Mutation Gene Biobank (NHMGB), the genetic biobank of Cardiomyology and Medical Genetics of the University of Campania Luigi Vanvitelli, which is a member of EuroBioBank and the Telethon Network of Genetic Biobanks (TNGB). All patients and healthy controls were not affected by ongoing infections and were not immunosuppressed at the time of bleeding, and did not perform any physical activity before the blood collection, since it has been demonstrated that in response to exercise, some cytokines (e.g., IL-6, TNF-α) are released by immune and muscle cells [14,15,16]. Peripheral blood was collected in Greiner Bio-One VACUETTE™ Z Serum Sep Clot Activator Tubes (Thermo Fisher Scientific, Waltham, MA, USA), centrifuged at 3000 rpm for 10 min at room temperature. The serum, transferred in cryogenic vials, was immediately stored in liquid nitrogen pending assays.

### 2.3. Cytokine, Chemokine and Growth Factor Quantification

A Bio-Plex Pro^TM^ Human Cytokine 27-plex Immunoassay 96-well kit (Bio-Rad Laboratory, Hercules, CA, USA) was used to measure the serum concentration of pro- and anti-inflammatory cytokines, chemokines and growth factors in the first group of patients. The kit includes: gamma interferon (IFN-γ), interleukin-1β (IL-1β), IL-1 receptor antagonist (IL-1ra),interleukin-2 (IL-2), IL-4, IL-5, IL-6, IL-7, IL-8, IL-9, IL-10, IL-12 (p70), IL-13, IL-15, IL-17, tumor necrosis factor-alpha (TNF-α), interferon gamma-induced protein 10 (IP-10), monocyte chemoattractant protein-1 (MCP-1), macrophage inflammatory protein-1alpha (MIP-1α), MIP-1β, RANTES, platelet-derived growth factor-BB (PDGF-BB), basic fibroblast growth factor (bFGF), eotaxin, granulocyte colony-stimulating factor (G-CSF), granulocyte-macrophage colony-stimulating factor (GM-CSF), and vascular endothelial growth factor (VEGF). The three isoforms of TGF-β were detected in the same serum samples using a Bio-Plex Pro^TM^ TGF-β 3-plex assay (Bio-Rad Laboratory). The second group of patients was tested using a Bio-Plex Pro^TM^ Human Inflammation panel 1 24-plex (Bio-Rad Laboratory) to detect the serum concentration of tumor necrosis factor (TNF) related molecules (APRIL/TNFSF13, BAFF/TNFSF13B, sCD30/TNFRSF8, sCD163, chitinase 3-like 1, glycoprotein-130 (gp130/sIL-6Rβ), IFN-β, IL-11, IL-19, IL-20, IL-26, IL-27 (p28), IL-28A/IFN-λ2, IL-29/IFN- λ1, IL-32, IL-34, IL-35, LIGHT/TNFSF14, osteocalcin, pentraxin-3, sTNF-R1, sTNF-R2, TSLP, TWEAK/TNFSF12). The assays were performed according to the manufacturer’s guidelines and the plates were read on the BioPlex 200 system (Bio-Rad), powered by Luminex xMAP technology.

The serum of each patient and healthy control was diluted 1:4 and tested in duplicate; sera from patients and healthy controls was always mixed in all plates to reduce the variability. Data are expressed as a concentration (pg/mL). The concentration of analyte bound to each bead was proportional to the median fluorescence intensity (MFI) of the reporter signal and was corrected by the standards provided in the kit (Bio-Rad). 

### 2.4. Statistical Analysis

A description of the participant characteristics at baseline was provided in terms of absolute number and percentages for categorical data and means with standard deviations (SDs) and medians with value ranges for continuous data. A Mann–Whitney test was used to assess the differences in cytokine levels between the Musc-LMNA patients and controls, Musc-LMNA and mutated LMNA patients, and Musc-LMNA patients and those with other muscular disorders. A nonparametric K-sample test on the equality of medians within groups was used to assess the differences in cytokine levels among different Musc-LMNA subgroups (i.e., myopathy, cardiomyopathy and myopathy plus cardiomyopathy). Receiver operating characteristic (ROC) curves were used to estimate the diagnostic potential of the quantified individual cytokines to discriminate between groups. Statistical analyses were performed using STATA statistical software, version 15 (StataCorp. 2017. Stata Statistical Software: Release 15. College Station, TX: StataCorp LLC) and R software environment for statistical computing and graphics (https://www.r-project.org/).

## 3. Results

### 3.1. Evaluation of Cytokine Levels in the Sera of the Musc-LMNA Patients (n = 40) and Comparison with Those of Healthy Controls

**Figure 1:**
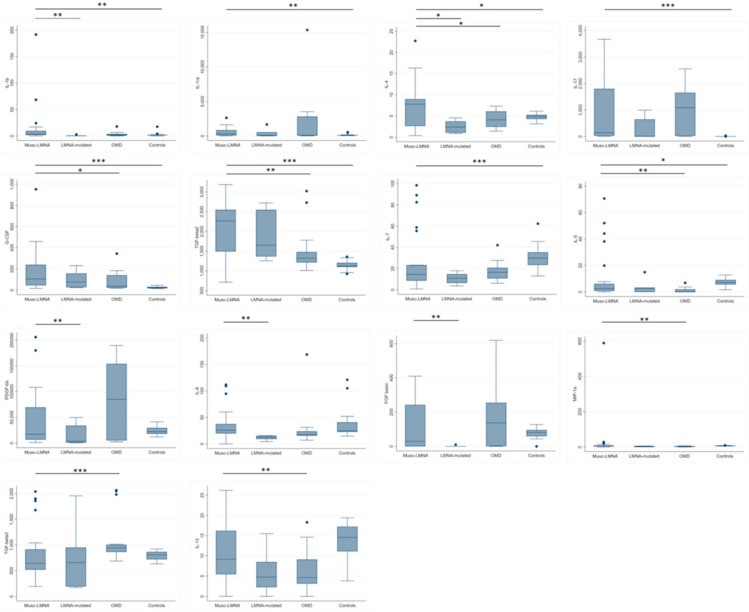
The immune mediator expression in serum samples from Musc-LMNA and LMNA-mutated patients, patients with other muscular disorders (OMD) and healthy controls. Among the 30 immune mediators analyzed, the serum concentration levels of six molecules (interleukin-1β (IL-1β), *p* = 0.0024; IL-1 receptor antagonist (IL-1ra), *p* = 0.0066; IL-4, *p* = 0.0314; IL-17, *p* < 0.0001; granulocyte colony-stimulating factor (G-CSF), *p* < 0.0001; TGF-β2, *p* < 0.0001) were significantly higher in the Musc-LMNA patients than in healthy controls. IL-5 (*p* = 0.0029) and IL-7 (*p* = 0.0011) levels were significantly decreased in the Musc-LMNA patients compared to controls. IL-1β (*p* = 0.0032), IL-4 (*p* = 0.0120), platelet-derived growth factor-BB (PDGF-BB (*p* = 0.0161)), IL-8 (*p* = 0.0038) and basic fibroblast growth factor (bFGF (*p* = 0.0041)) were higher in the Musc-LMNA patients compared to the LMNA-mutated patients. In the comparison between Musc-LMNA and OMD groups, IL-4 (*p* = 0.0309), G-CSF (*p* = 0.0194), TGF-β2 (*p* = 0.0033), IL-5 (*p* = 0.0080), macrophage inflammatory protein-1alpha (MIP-1α (*p* = 0.0120)) and IL-13 (*p* = 0.0147) were higher in Musc-LMNA, whereas TGF-β3 (*p* = 0.0007) was lower in this group. Dark horizontal lines represent the means, with the box representing the 25th and 75th percentiles, and the whiskers representing the 5th and 95th percentiles. * *p* < 0.05; ** *p* < 0.01; *** *p* < 0.001.

**Figure 2:**
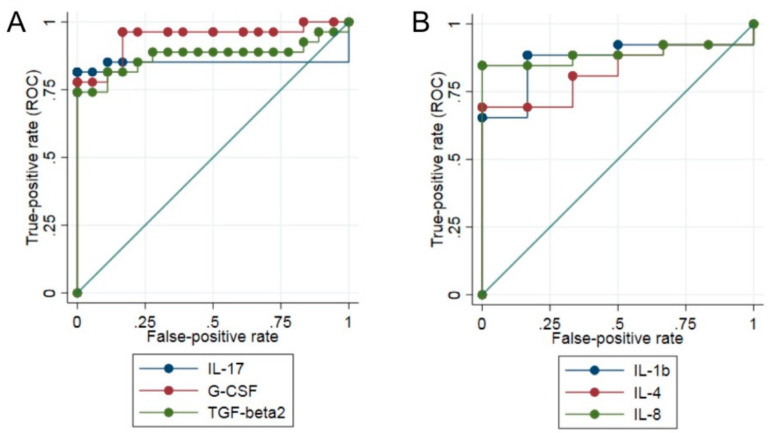
ROC analysis of the serum levels of IL-17, G-CSF and TGF-b2 for the comparison between the Musc-LMNA patients and controls (**A**) and of IL-1b, IL-4 and IL-8 between Musc-LMNA and LMNA-mutated groups (**B**). The true positive rate (sensitivity (SE)) on the y-axis is represented as a function of the false positive rate (100%—specificity% (SP)) on the x-axis. Higher y values correspond to a higher SE, and lower x values correspond to a higher SP.

The quantification of the serum concentration of 27 inflammatory cytokines showed a significant increase in the levels of IL-1b (*p* = 0.0024), IL-1ra (*p* = 0.0066), IL-4 (*p* = 0.0314), IL-17 (*p* < 0.0001), G-CSF (*p* < 0.0001), and TGF-b2 (*p* < 0.0001) in the Musc-LMNA group when compared to the healthy controls (Table 2 and Figure 1). The presence of IL-7 and IL-5 was significantly lower in laminopathic patients than in the controls (*p* = 0.0011 and *p* = 0.0244, respectively (Table 2 and Figure 1)). ROC curves for IL-17, G-CSF and TGF-β2, the most significantly dysregulated cytokines in the Musc-LMNA patients, are presented in Figure 2A. The area under the ROC curve (AUC) was 0.85 for IL-17, 0.94 for G-CSF, and 0.87 for TGF-b2, suggesting that the measurement of the levels of these cytokines might allow us to discriminate between Musc-LMNA and healthy controls.

### 3.2. IL-1β, IL-4 and IL-8 Levels in Musc-LMNA Compared to LMNA-Mutated Patients

Overall, 10 cytokines were found to be significantly higher in the Musc-LMNA patients compared to the group of laminopathic patients without an apparent muscle involvement (LMNA-mutated): PDGF-BB (*p* = 0.0161), IL-1β (*p* = 0.0032), IL-4 (*p* = 0.0120), IL-8 (*p* = 0.0038), and bFGF (*p* = 0.0041 (Table 2 and Figure 1)). ROC curves for IL-1β, IL-4 and IL-8 are reported in Figure 2B. For IL-1β, IL-4 and IL-8 the AUC was 0.87, 0.82 and 0.88, respectively. These findings may be relevant to the understanding of pathogenetic pathways specifically involved in non-muscular laminopathy.

### 3.3. Differential Expression of Cytokines between Patients Affected by Musc-LMNA and Other Muscular Disorders

The comparison between the Musc-LMNA patients and the group including patients affected by other muscular disorders demonstrated a significant increase in laminopathic patients of IL-4 (*p* = 0.0309), IL-5 (*p* = 0.0080), IL-13 (*p* = 0.0147), G-CSF (*p* = 0.0194), MIP-1α (*p* = 0.0120) and TGF-β2 (*p* = 0.0033 (Table 2 and Figure 1)). TGF-β3 was observed at significantly lower levels in the Musc-LMNA patients than in the other muscular disorders group (*p* = 0.0007 (Table 2 and Figure 1)).

### 3.4. IL-17 Is Significantly Down-Regulated in Musc-LMNA Patients with Also Cardiac Involvement

The Musc-LMNA patients were classified according to the presence or absence of cardiac involvement. No significant difference among these subgroups was observed by cytokine quantification with the exception of IL-17; the expression levels of this cytokine were significantly lower in laminopathic patients with muscular and cardiac symptoms (*p* = 0.010 (Table 3)). 

### 3.5. Analysis of TNF-α-Related Cytokines in Sera of Musc-LMNA Patients and Healthy Controls

**Figure 3:**
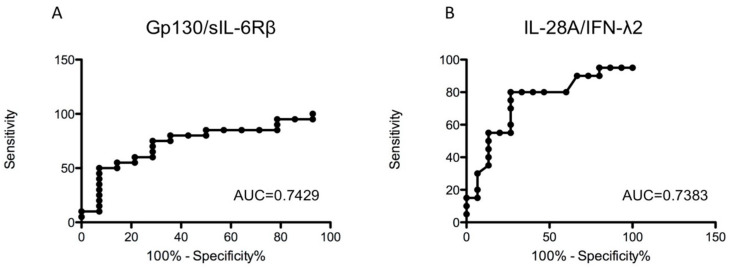
Areas under the curves obtained with the sensitivity and specificity for the serum levels of glycoprotein-130 (gp130/sIL-6Rb) (**A**) and IL-28A/IFN-l2 (**B**) in Musc-LMNA. True positive rate (sensitivity (SE)) on the y-axis is represented as function of the false positive rate (100%—specificity% (SP)) on the x-axis. Higher y values correspond to a higher SE, whereas lower x values correspond to a higher SP.

As previously demonstrated, endosomal Toll-like receptors (TLRs) and the TNF-α pathway play a key role in the pathogenic mechanisms of Musc-LMNA [6]. In order to evaluate potential biomarkers, among the wide group of TNF-α-related cytokines, to discriminate between Musc-LMNA and healthy controls, sera from a further 13 Musc-LMNA patients and 15 healthy controls were analyzed by a multiplex immunoassay specific for TNF pathways (Table 4). Among the 24 tested molecules, the signal-transducing molecule gp130/sIL-6Rβ and IL28A/IFN-λ2 were significantly dysregulated in the Musc-LMNA patients compared to the healthy controls (*p* = 0.0149 and *p* = 0.0147, respectively). The ROC curves for these proteins are reported in Figure 3. For gp130/sIL-6Rβ, the AUC was 0.7429, and for IL28A/IFN-λ2 0.7383.

## 4. Discussion

In the present study, the serum levels of IL-1β, IL-1ra, IL-4, IL-17, G-CSF and TGF-β2 were significantly higher in the Musc-LMNA patients compared to healthy controls, whereas IL-7 and IL-5 concentrations were significantly lower in the Musc-LMNA patients than in controls. 

A consistent increase in both pro- and anti-inflammatory cytokines in the Musc-LMNA patients is not surprising. The involvement of the immune response, and in particular of the innate immunity in the pathogenic mechanisms of LMNA-related myopathies, has been previously demonstrated [7,17]. The prevalence of macrophages among a few mononuclear immune cells observed in laminopathic muscle tissue may explain the high concentration of TGF-β2 measured in this work and other studies. TGF-β2 is a multi-functional cytokine that regulates cell proliferation, differentiation, migration and survival. It plays a critical role in development, wound healing and immune responses through its regulatory effects on many cell types, including epithelial and hematopoietic cells [18]. This cytokine plays a critical biological role in promoting the alternative activation of macrophages into the M2 subtype, which are considered to be anti-inflammatory cells [18].

M2 macrophages can be pathogenic as they participate in extracellular matrix (ECM) remodeling on tissues wounded by acute and chronic inflammatory stimuli [19]. The persistent activation of M2 macrophages has been thought to contribute highly to myofiber necrosis and fibrosis, promoting fibroblast proliferation and connective tissue deposition [17,20], which may explain to some extent the activation of fibrotic processes in cardiac and skeletal muscle often observed in laminopathic patients [17]. The hypothesis that M2 macrophages are prevalent in Musc-LMNA patients may be further supported by the significant increase in serum IL-1ra and IL-4 observed in these patients compared to controls. 

IL-17 is a pleiotropic cytokine with a pro-inflammatory function that induces the production of other downstream inflammatory cytokines and chemokines [21]. It is produced by various inflammatory cells, including T cells and macrophages, and modulates the expression of several pro-inflammatory molecules (TNF-α, IL-1β, and CCL2 (MCP-1)) and matrix metalloproteinases [22], as opposed to anti-inflammatory cytokines and chemokines such as G-CSF and IL-8 [19]. Furthermore, IL-17 may indirectly induce macrophage polarization towards the M2 phenotype [23]. The increase in Musc-LMNA patients’ sera may correlate with the prevalence of macrophages among immune cells infiltrating laminopathic skeletal muscle tissue.

G-CSF is a cytokine that stimulates the survival, proliferation, differentiation and function of neutrophil precursors and mature neutrophils via its receptor G-CSFR [24]. It has also been reported to modulate inflammatory responses, modulating monocyte differentiation into M2 anti-inflammatory macrophages [24]. As for TGF-β2 and IL-17, the high concentration of G-CSF in Musc-LMNA patients’ serum may be accounted for, to some extent, due to the presence of macrophages in laminopathic muscle tissue. The diagnostic value of TGF-β2, IL-17 and G-CSF in the Musc-LMNA patients’ serum was examined by ROC curve analysis. Promising results were obtained for all three cytokines, particularly for G-CSF (0.94), which make these proteins potentially important diagnostic biomarkers.

IL-7 is a non-redundant cytokine which plays a key role in T-cell development and function [25]. It is important for early T-cell development as well as for T-cell homeostasis; it is secreted by stromal cells in the thymic and bone marrow environment [25]. Considering the IL-7 signaling pathway, it is reasonable to hypothesize that the significant decrease in this cytokine observed in the Musc-LMNA patients’ sera may be related to the chronic production of TGF-β2. Indeed, it was demonstrated that IL-7 and TGF-β have opposing effects in the inflammatory process and fibrosis [26,27]. Furthermore, it has been shown that a blockade of IL-7 signaling favors the generation of M2 phenotype macrophages by affecting the cytokine production of T helper-1 (Th1) and T helper-2 (Th2) cells [28]. Therefore, it is possible that the prevalence of M2 macrophages in Musc-LMNA muscle tissue can negatively modulate IL-7 production. 

Comparing Musc-LMNA to non-muscular laminopathies, it has been found that a significant increase in the concentration of PDGF-BB, IL-1β, IL-4, IL-8, and bFGF in the Musc-LMNA patients’ sera. The highest levels were observed for IL-1β, IL-4 and IL-8. The meaning of the different concentrations of these three cytokines is not clear: non-muscular laminopathies, in fact, are often characterized by inflammatory processes. The diagnostic value of these three cytokines was examined by ROC curve analysis; based on this analysis, the cited cytokines might be considered good candidate biomarkers with a high specificity for Musc-LMNA, and deserve further investigation to understand whether their expression levels might contribute to the different phenotypes observed between Musc-LMNA and non-muscular laminopathies. 

The characterization of cytokine profiles among laminopathic patients with or without cardiac involvement did not show any significant difference in cytokine serum concentrations, except for IL-17. Levels of IL-17 were significantly lower in laminopathic patients with the co-existence of myopathic and cardiac symptoms. It is important to keep in mind that the onset of cardiomyopathy might precede the onset of myopathy and vice versa. Therefore, it would be interesting to understand the biological significance of different IL-17 secretion in the onset of the two phenotypes from further investigations. 

Considering the prevalence of macrophages in Musc-LMNA muscle tissue and the high serum concentration of cytokines related to the M2 macrophagic subgroup, in the present study the serum levels of 24 molecules of the TNF-α signaling pathway was also evaluated. The IL28A/IFNλ2 level has been shown to be significantly higher in Musc-LMNA patients than in healthy controls. This result was particularly intriguing: IL-28A is a cytokine with antiviral, antitumour and immunomodulatory activities, playing a critical role in the Toll-like receptor (TLR) induced antiviral defense, predominantly in the epithelial tissues. Recently, the involvement of endosomal TLRs in the pathogenic mechanisms of Musc-LMNA has been demonstrated [7]. It will be of interest to further investigate the role of IL28A/IFNλ2 in Musc-LMNA pathogenesis. 

Glycoprotein-130 (gp130), the central signal transducer of IL-6-related cytokines, is widely expressed in the mammalian organism, including the developing and adult heart [29]. Gp130 is a cytokine involved in various inflammatory and immunoregulatory mechanisms [30,31] and plays an essential role in cardiac development, potentially by regulating cardiomyogenesis, cardiomyocyte survival and growth [32]. Indeed, balanced gp130 signaling appears to be cardioprotective, whereas altered serum levels of this cytokine contribute to maladaptation and heart failure. A substantial decrease in gp130 does not alter cardiac function or structure, but it makes the heart highly susceptible to every form of stress and promotes cardiac dysfunction [33,34,35]. Therefore the low levels of gp130 in Musc-LMNA patients might contribute to cardiac dysfunction caused by LMNA mutations that might be considered stress factors. 

## 5. Conclusions

Genetic analysis and clinical examination remain the most important factors in the diagnosis of Musc-LMNA. However, the ability to distinguish between Musc-LMNA patients and healthy controls or between Musc-LMNA patients and those with non-muscular laminopathies, based on their serum cytokine levels, would be of great value in clinical practice. According to the results of this study, TGF-β2, IL-17 and G-CSF might be considered as potential biomarkers in order to discriminate between a form of muscle-related laminopathy and a healthy subject. While involvement of TGF-β2 in musc-LMNA pathogenetic pathways, causing activation of profibrotic signaling and fibrotic processes, has been proven in human and mouse experimental models [17], the contribution of IL-17 and G-CSF to disease onset or progression has not been explored and warrants investigation. Moreover, the identification of a differential expression of IL-1β, IL-4 and IL-8 in relation to the different forms of laminopathy might provide a new hint for the understanding of the pathogenic mechanisms underlying the diverse phenotypes caused by LMNA mutations, while specific analysis of the secretome of HGPS, MADA and FPLD2 will identify relevant pathogenetic pathways for those diseases.

We are aware that confirmatory studies with a larger number of patients and longitudinal evaluations are required to prove reliability of these biomarkers in the diagnosis and follow-up of skeletal muscle laminopathies.

## Figures and Tables

**Table 1:** Clinical data of patients included in the study.

	*Musc-LMNA 1st Group* (*n* = 40)	*Musc-LMNA 2nd Group* (*n* = 13)	*LMNA*-Mutated (*n* = 10)	Other Muscular Diseases OMD (*n* = 22)	Healthy Controls *1st Group* (*n* = 22)	Healthy Controls *2nd Group* (*n* = 15)
Age at time of blood collection (mean ± SD years)	43.76 ± 16.80	49.18 ± 12.86	36.17 ± 15.41	32.5 ± 14.24	30.73 ± 7.99	41.25 ± 7.94
Gender (F/M)	15/25	6/7	5/5	3/19	19/3	9/6
Skeletal muscle Disease duration (mean ± SD years)	25 ± 14.2	25.19 ± 15.22	7.7 ± 1.5	14.6 ± 13	-	-
Cardiac involvement (age of onset)	31.15 ± 12.26	24.65 ± 16.58	28.67 ± 10.97	18.63 ±14.60	-	-

First group: group of patients tested for the expression of 27 inflammatory cytokines, chemokines and growth factors; 2nd group: group of patients tested for additional tumor necrosis factor (TNF)-related cytokines.

**Table 2:** Serum concentration of pro- and anti-inflammatory cytokines, chemokines and growth factors in patients and controls.

	Musc-*LMNA* (*n* = 40)	*LMNA*-Mutated (*n* = 10)	Other Muscular Disorders (*n* = 22)	Healthy Controls (*n* = 22)
**PDGF-BB**	**17,315.96 (1161.56–205,948.70)**	**3697.91 (686.09–49,690.25)**	84,812.43 (2505.20–189,606.70)	22,222.5 (12,030.51–41,331.11)
**IL-1b**	**3.73 (0–191.50)**	**0.15 (0–2.85)**	2.145 (0–17.73)	**1.69 (0–17.41)**
**IL-1ra**	**294.61 (0–2616.60)**	70.43 (0–1659.02)	98.27 (4.49–15,348.96)	**75.39 (40.99–543.56)**
**IL-2**	**9.67 (0–82.13)**	**0 (0–0.79)**	11.87 (0–32.81)	1.70 (0–24.23)
**IL-4**	**7.77 (0.37–22.73)**	**2.40 (0.89–4.54)**	**4.10 (1.44–7.33)**	**4.86 (3.13–6.13)**
**IL-5**	**2.62 (0–70.53)**	2.84 (0–14.91)	**0 (0–6.85)**	**7.23 (1.64–12.77)**
**IL-6**	**10.14 (0–153.35)**	**2.81 (0.31–11.75)**	6.77 (2.96–52.31)	9.65 (4.90–64.69)
**IL-7**	**14.43 (0.74–98.53)**	10.76 (3.57–17.86)	16.48 (6.13–42.03)	**29.87 (13.00–62.31)**
**IL-8**	**25.79 (0–111.64)**	**12.10 (4.31–15.56)**	17.47 (7.14–168.52)	24.53 (14.64–120.83)
**IL-9**	**32.62 (0–143.24)**	**0 (0–53.83)**	33.27 (0–286.82)	23.22 (10.99–60.36)
IL-10	12.28 (1.01–175.74)	9.20 (1.12–188.78)	9.72 (3.72–57.47)	8.53 (3.95–62.91)
IL-12(p70)	42.40 (0–139.44)	5.70 (1.39–99.16)	16.78 (5.06–79.27)	34.23 (13.46–153.09)
**IL-13**	**9.16 (0–26.20)**	4.77 (0–15.50)	**4.64 (0–18.29)**	14.57 (3.83–19.42)
IL-15	0 (0–37.61)	0 (0–0)	0 (0–0)	0 (0–0)
**IL-17**	**137.84 (0–3670.05)**	0.17 (0–993.46)	1088.45 (0–2552.44)	**0 (0–26.37)**
Eotaxin	201.02 (0–1470.63)	134.36 (0–447.23)	666.84 (2.25–3051.93)	248.46 (109.92–1141.27)
**FGF basic**	**29.21 (0–409.01)**	**0 (0–9.98)**	136.77 (0–618.20)	81.19 (0–127.15)
**G-CSF**	**105.17 (16.07–951.87)**	76.42 (23.21–230.76)	**36.53 (17.14–344.58)**	**25.07 (14.09–44.62)**
GM-CSF	0 (0–2603.30)	0 (0–621.94)	0 (0–2971.71)	0 (0–112.41)
IFN-g	284.82 (0–2380.49)	138.48 (0–462.36)	229.58 (0–600.53)	177.33 (84.78–437.35)
IP-10	1353.57 (456.85–11,728.81)	1979.34 (410.53–103,596.80)	5276.79 (477.36–106,593.55)	1703.98 (1030.39–3787.98)
MCP-1 (MCAF)	152.44 (0–2333.73)	43.82 (14.97–258,031.90)	163.23 (0–2972.09)	74.98 (28.88–327.95)
**MIP-1a**	**6.34 (0–588.43)**	3.33 (0–5.77)	**3.08 (0–8.04)**	6.61 (5.32–9.72)
**MIP-1b**	**139.07 (76.69–505.49)**	**78.12 (51.70–167.59)**	224.45 (57.65–1002.74)	155.91 (83.62–302.21)
TNF-a	42.81 (5.48–423.51)	38.92 (15.17–215.62)	25.84 (9.51–61.89)	35.45 (21.01–63.35)
VEGF	114.81 (0–522.14)	2.12 (0.03–360.84)	48.05 (0–468.59)	234.62 (45.76–488.52)
TGF-b1	92,399.89 (6544.34–205,045.20)	64,905.52 (11,824.81–171,893.50)	104,118.90 (38,641.36–133,449.20)	72,071.24 (42,398.29–105,300.30)
**TGF-b2**	**2265.20 (712.94–3186.47)**	1651.26 (1255.47–2722.26)	**1317.43 (1008.99–3022.97)**	**1126.59 (922.55–1356.60)**
**TGF-b3**	**642.18 (190.97–2039.26)**	656.13 (173.47–1956.91)	**943.61 (686.71–2065.09)**	809.13 (632.75–921.18)

Values, expressed in pg/mL, are reported as the median (range). A Mann–Whitney test has been used to evaluate the statistical significance for muscular laminopathy (Musc-LMNA) patients versus controls, Musc-LMNA versus LMNA-mutated patients, and Musc-LMNA patients versus patients with other muscular disorders. Molecules whose expression levels were statistically significant (*p* < 0.05) have their values written in bold. RANTES cytokine, included in the 27-plex Immunoassay, has been excluded from the statistical analyses since most of the values were out of range, both for pathological samples and control samples.

**Table 3:** Cytokine profile in Musc-LMNA subgroups.

	Group			
	Myopathy	Cardiomyopathy	Myopathy + Cardiomyopathy	
	(*n* = 13)	(*n* = 11)	(*n* = 16)	*p*-Value *
	Median (Range)	Median (Range)	Median (Range)	
PDGF-BB	30,581.53 (5061.38–180,068.80)	20,489.26 (4599.65–205,948.70)	9564.63 (1161.56–68,897.70)	0.348
IL-1β	4.53 (0–191.50)	3.65 (0.82–24.07)	3.62 (0.04–16.90)	0.197
IL-1ra	710.35 (0–2616.60)	294.61 (74.82–998.68)	137.68 (7.30–1062.62)	0.191
IL-2	0.01 (0–82.13)	9.94 (0–52.66)	9.74 (0–40.63)	0.776
IL-4	7.24 (0.37–13.56)	7.19 (2.10–22.73)	7.97 (1.18–12.99)	0.948
IL-5	2.30 (0–51.97)	1.39 (0–70.53)	3.90 (0–38.08)	0.462
IL-6	7.91 (0–72.42)	10.42 (4.25–153.35)	10.69 (3.94–43.94)	0.348
IL-7	18.01 (0.74–98.53)	14.43 (6.88–89.10)	12.08 (5.34–55.48)	0.191
IL-8	23.12 (0–58.94)	28.91 (16.6–111.64)	25.93 (14.05–94.50)	0.670
IL-9	1.46 (0–111.50)	59.11 (0–143.24)	42.59 (0–109.43)	0.443
IL-10	12.10 (1.01–175.74)	13.47 (5.39–76.50)	11.66 (2.07–33.29)	0.776
IL-12(p70)	35.44 (1.18–114.07)	46.56 (11.63–134.04)	46.92 (0–139.44)	1.000
IL-13	7.73 (0–23.04)	9.42 (4.17–26.20)	11.76 (4.66–18.27)	0.104
IL-15	0 (0–12.33)	0 (0–37.61)	0 (0–0)	0.203
**IL-17**	831.73 (0–3670.05)	353.31 (3.98–2580.59)	**69.96 (0–1103.82)**	**0.010**
Eotaxin	302.70 (5.50–1470.63)	429.69 (15.94–1462.28)	81.01 (0–595.95)	0.072
FGF-b	85.05 (0–372.76)	117.38 (0–409.01)	25.79 (0–271.26)	0.776
G-CSF	200.57 (44.89–459.80)	239.16 (31.51–951.87)	44.64 (16.07–235.61)	0.070
GM-CSF	0 (0–2603.30)	0 (0–66.49)	0 (0–0)	0.085
IFNγ	356.67 (0–1462.33)	350.35 (0–2380.49)	252.11 (0–1171.02)	0.776
IP-10	2031.15 (492.37–11,728.81)	1278.70 (705.29–3133.58)	1187.12 (456.85–4185.00)	0.104
MCP-1(MCAF)	288.82 (34.20–1358.13)	132.90 (34.11–1567.18)	111.38 (0–2333.73)	0.438
MIP-1α	7.23 (2.36–588.43)	4.01 (0.85–27.97)	4.39 (0–18.84)	0.462
MIP-1β	159.00 (90.58–505.49)	158.68 (88.43–391.97)	95.06 (76.69–207.82)	0.124
TNF-α	52.56 (5.48–360.57)	42.81 (27.39–423.51)	37.19 (12.44–188.31)	0.635
VEGF	52.54 (0.63–521.33)	193.47 (22.18–522.14)	106.01 (0–390.42)	0.776
TGF-β1	92,399.89 (6544.34–205,045.20)	109,007.20 (37,750.73–168,194.40)	74470.68 (27,310.81–178,799.60)	0.435
TGF-β2	2335.85 (712.94–3094.08)	2252.80 (1303.16–2786.12)	2342.04 (1222.87–3186.47)	0.179
TGF-β3	560.83 (190.97–989.91)	541.31 (357.53–2039.26)	697.98 (306.96–1901.91)	0.472

* Nonparametric K-sample test on the equality of medians within groups of Musc-LMNA (i.e., myopathy versus cardiomyopathy and versus myopathy + cardiomyopathy). In bold is highlighted the cytokine whose expression levels in the myopathy + cardiomyopathy group are significantly the lowest.

**Table 4:** Serum concentration of TNF-α-related cytokines in Musc-LMNA and controls.

Cytokine	Musc-*LMNA* (*n* = 20)	Controls (n = 15)	*p*-Value
APRIL/TNFSF13	25,801.60 ± 22,051.82	21,467.51 ± 12,355.35	0.8705
BAFF/TNFSF13B	15.32 ± 3.65	14.08 ± 3.64	0.3941
sCD30/TNFRSF8	610.56 ± 232.31	618.21 ± 285.94	0.8705
sCD163	189,251.81 ± 84,913.40	178,724.79 ± 114,129.78	0.376
Chitinase 3-like 1	16,470.27 ± 4435.62	16,516.77 ± 3408.92	0.4211
**gp130/sIL-6Rβ**	**39,283.66 ± 5520.68**	43,765.82 ± 7997.48	**0.0149**
IFN-β	22.31 ± 5.69	21.94 ± 4.64	0.8577
IL-11	1.51 ± 0.52	1.41 ± 0.71	0.2927
IL-19	76.52 ± 13.22	74.75 ± 13.96	0.5344
IL-20	40.35 ± 13.87	36.24 ± 8.56	0.3163
IL-26	13.72 ± 4.97	15.01 ± 5.78	0.5776
IL-27 (p28)	0.0 ± 0.0	0.0 ± 0.0	-
**IL-28A/IFN-λ2**	**21.10 ± 3.32**	18.25 ± 2.68	**0.0147**
IL-29/IFN-λ1	12.49 ± 3.63	13.53 ± 4.28	0.4815
IL-32 ^1^	5.90 ± 5.61	10.43 ± 4.22	0.1516
IL-34 ^2^	74.33 ± 53.86	60.47 ± 23.51	0.5812
IL-35	112.27 ± 47.50	111.80 ± 57.47	0.8962
LIGHT/TNFSF14 ^3^	47.65 ± 41.68	49.12 ± 37.57	0.9776
Osteocalcin	4550.45 ± 1715.57	5580.24 ± 1975.88	0.1107
Pentraxin-3	1170.04 ± 375.80	1191.73 ± 571.97	0.7443
sTNF-R1	6198.05 ± 4290.42	5440.41 ± 1320.81	0.7443
sTNF-R2	10,766.25 ± 8250.28	10,217.52 ± 6429.89	0.7691
TSLP	55.30 ± 22.31	51.67 ± 26.24	0.4029
TWEAK/TNFSF12	766.58 ± 187.24	796.98 ± 178.46	0.7691

Values, expressed in pg/mL, are reported as mean ± standard deviation. A Mann–Whitney test has been used to evaluate the statistical significance for the Musc-LMNA patients versus controls. Those molecules, whose expression levels were significantly different between the Musc-LMNA patients and controls, and their values are in bold. ^1^ Only 5 out of the 13 Musc-LMNA patients’ and 6 out of the 15 controls’ sera were tested. ^2^ Only 8 out of the 13 Musc-LMNA patients’ and 8 out of the 15 controls’ sera were tested. ^3^ Only 11 out of the 13 Musc-LMNA patients’ and 13 out of the 15 controls’ sera were tested.
